# Characteristics of children requiring admission to neonatal care and paediatric intensive care before the age of 2 years in England and Wales: a data linkage study

**DOI:** 10.1136/archdischild-2023-325986

**Published:** 2024-02-12

**Authors:** Sarah E Seaton, Cheryl Battersby, Peter J Davis, Alan C Fenton, Josie Anderson, Tim J van Hasselt, Elizabeth Draper

**Affiliations:** 1 Department of Population Health Sciences, University of Leicester, Leicester, UK; 2 Neonatal Medicine, School of Public Health, Imperial College London, London, UK; 3 Paediatric Intensive Care Unit, Bristol Royal Hospital for Children, Bristol, Bristol, UK; 4 Newcastle Neonatal Service, Newcastle Upon Tyne Hospitals NHS Foundation Trust, Newcastle Upon Tyne, UK; 5 Bliss Baby Charity, London, UK

**Keywords:** Intensive Care Units, Paediatric, Intensive Care Units, Neonatal, Epidemiology

## Abstract

**Objective:**

To quantify the characteristics of children admitted to neonatal units (NNUs) and paediatric intensive care units (PICUs) before the age of 2 years.

**Design:**

A data linkage study of routinely collected data.

**Setting:**

National Health Service NNUs and PICUs in England and Wales

**Patients:**

Children born from 2013 to 2018.

**Interventions:**

None.

**Main outcome measure:**

Admission to PICU before the age of 2 years.

**Results:**

A total of 384 747 babies were admitted to an NNU and 4.8% (n=18 343) were also admitted to PICU before the age of 2 years. Approximately half of all children admitted to PICU under the age of 2 years born in the same time window (n=18 343/37 549) had previously been cared for in an NNU.

The main reasons for first admission to PICU were cardiac (n=7138) and respiratory conditions (n=5386). Cardiac admissions were primarily from children born at term (n=5146), while respiratory admissions were primarily from children born preterm (<37 weeks’ gestational age, n=3550). A third of children admitted to PICU had more than one admission.

**Conclusions:**

Healthcare professionals caring for babies and children in NNU and PICU see some of the same children in the first 2 years of life. While some children are following established care pathways (eg, staged cardiac surgery), the small proportion of children needing NNU care subsequently requiring PICU care account for a large proportion of the total PICU population. These differences may affect perceptions of risk for this group of children between NNU and PICU teams.

**What is already known on this topic?**

- Some children, for example, those born very preterm, admitted to a neonatal unit (NNU) following birth are at increased risk of needing further healthcare in early life and beyond.
- No study has quantified the number or percentage of children who require care in an NNU and a paediatric intensive care unit (PICU) in the first 2 years.

**What this study adds?**

- Few children admitted to NNU require PICU admission before two years, but half of PICU admissions before two years have received care in an NNU
- The main reasons for first PICU admission are cardiac conditions (primarily term born children) and respiratory conditions (primarily emergency admissions from children born preterm)

**How this study might affect research, practice or policy**

- The healthcare professionals caring for babies and children in NNUs and PICUs see some of the same children and closer collaboration is essential.

## Background

Newborn babies requiring specialist care are admitted to neonatal units (NNUs), with admissions generally taking place shortly after birth and before discharge home. Reasons for admission include prematurity, congenital anomalies, respiratory difficulties and infection, and there has been improved survival of the most vulnerable babies.^1–3^ Most babies will have short NNU stays, but those with more complex conditions or born extremely preterm can have prolonged stays.^4^ For babies who reach term-corrected age and continue to require ongoing hospital care, transfer to paediatric care is an option, but this differs across the UK. There are 169 NNUs and 24 paediatric intensive care units (PICUs) across England and Wales. NNUs differ in their surgical and cardiac on-site provision, which may necessitate transfer to PICU in the neonatal period.

PICUs provide care for critically ill children until ~16 years in the UK. Children can be admitted from a variety of healthcare settings (eg, directly from an NNU or an emergency department). Around 40% of admissions to PICU are children aged under 1 year, accounting for ~50% of PICU bed days, but it is unknown how many of these children previously received care in an NNU. There may have been changes in the profile of children,^5 6^ and there are increasing numbers of children admitted to PICU with technology dependency and complex conditions, potentially following neonatal care.^7^

In the UK, data for all NNU admissions are available in the National Neonatal Research Database (NNRD). Likewise, all admissions to PICU are collected by the Paediatric Intensive Care Audit Network (PICANet). PICANet collects information about all care received in a PICU setting. These are mainly intensive care days but also a proportion of high-dependency care days provided in the PICU.^8^ This national data collection provides a unique opportunity to combine both databases to quantify at a population level; the characteristics of children previously cared for in NNU who are also admitted to a PICU. This is vital to share understanding between neonatal and paediatric clinical communities, inform resource needs and focus future research. In this work, we investigate the healthcare needs of children in their first 2 years of life, born from 2013 to 2018, focusing on those admitted to both NNU and PICU.

## Methods

### Population cohort

We used the NNRD and PICANet to create a linked record for each baby in the cohort up to the age of 2 years. We included two cohorts which we used as denominators for this work: (1) all babies born and admitted to an NNU (at least 1 day of intensive care; high dependency care or special care as defined by the British Association of Perinatal Medicine^9^) in the first week after birth in England or Wales between 1 January 2013 and 31 December 2018 and (2) all children who were born between 1 January 2013 and 31 December 2018 and admitted to PICU aged under 2 years between 2013 and 2020.

### Data sources

The NNRD holds data on the demographics, care and outcomes of babies admitted for neonatal care, created from information submitted by hospitals to a national electronic patient record system.^10^ PICANet holds information about the demographics, care and outcomes of children admitted to PICU, with data entry required within 3 months of the child’s discharge.^11^

Personal identifiers (NHS number, date of birth, surname, postcode) were provided by the NNRD and PICANet to NHS Digital (now NHS England) for all children born between 1 January 2013 and 31 December 2018. NHS Digital undertook data linkage across the cohorts to inform us of any children common to the NNRD and PICANet prior to the transfer of pseudoanonymised data to the study team. The completion rates of NHS numbers are known to be high in the NNRD and PICANet providing assurance that the linkage was as complete as possible.

### Statistical analysis

We investigated the neonatal characteristics of children by whether they were admitted to PICU and NNU or only an NNU. Due to anonymisation of data preventing the use of dates of admission/discharge, we estimated the care pathway of their first PICU admission as follows: (1) admitted to PICU during NNU stay: the child’s NNU stay was greater than the age on PICU admission; (2) directly transferred to PICU from NNU with no return to NNU: their age on admission to PICU was within ±1 day of their age on final discharge from neonatal care; (3) admission to PICU was after NNU: their age on PICU admission was greater than the total stay in NNU.

Information regarding PICU admission was categorised according to reason for the first PICU admission. Admission categories were formed from the four most common primary diagnoses: ‘cardiac’; ‘respiratory’; ‘gastrointestinal’ and ‘neurological’. A fifth category ‘other’ was created for all diagnoses which were not previously captured. We explored characteristics of the children by reason for first admission to PICU. We did this overall and by gestational age to investigate any relationship between prematurity and diagnosis.

We calculated the percentage of children and days of care provided in NNU from children cared for in NNU and PICU. We used the denominator of all care/children in the NNU outlined (1). Similarly, we calculated the percentage of children, admissions and days of care generated in PICU by those children previously cared for in an NNU. When doing this, we used a denominator of children/care in PICU outlined in (2).

To examine the care pathway of children previously cared for in an NNU who have multiple PICU admissions, we explored the reasons for first and subsequent admissions (up to the third admission) in the first 2 years of life using a Sankey diagram, where the width of each connection is relative to the number of admissions attributed to that clinical condition.

## Results

### Study cohort

**Figure 1:**
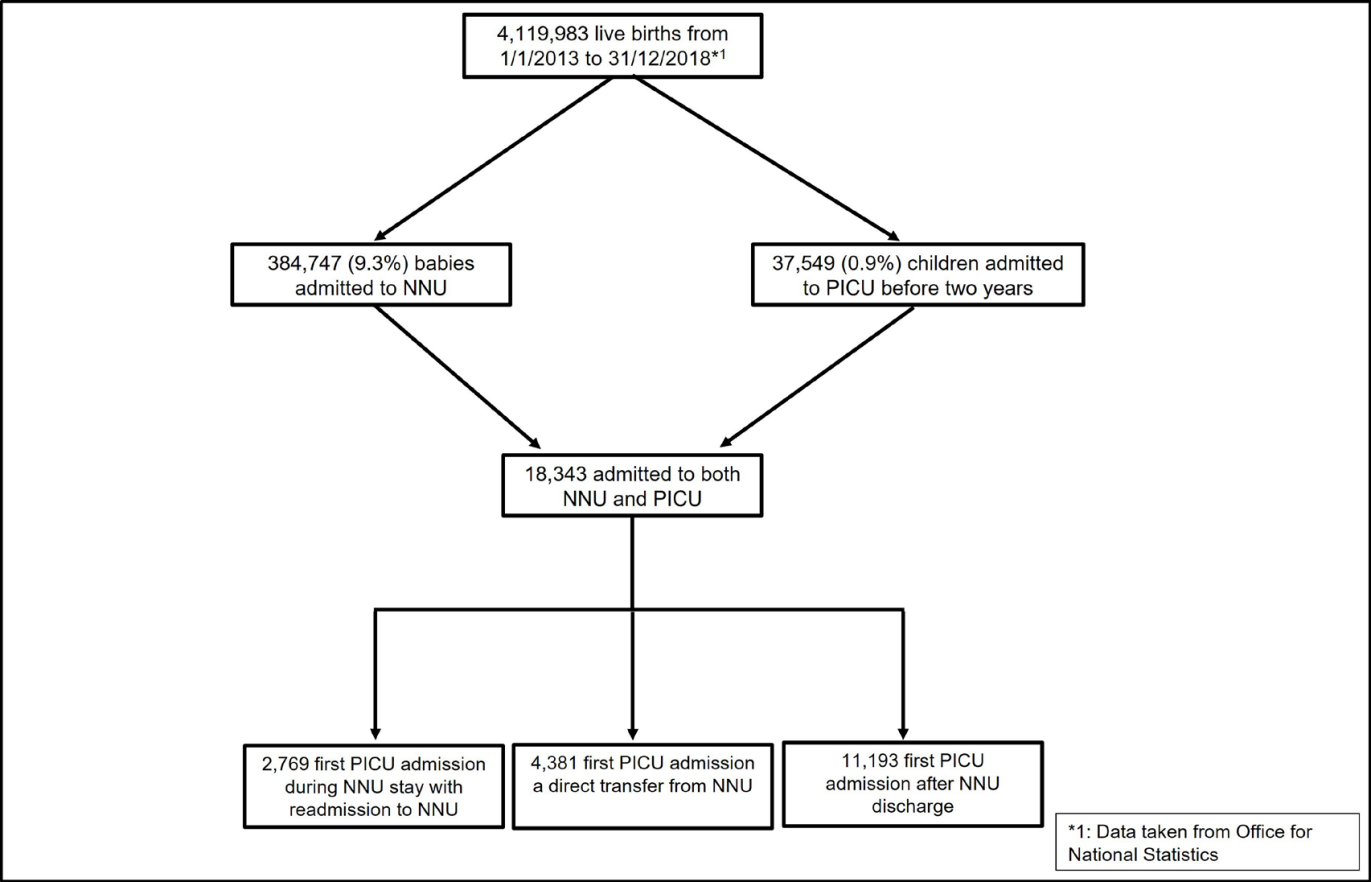
Flow chart showing the flow of the babies born from 2013 to 2018 and admitted to an NNU and/or PICU in England and Wales. NNU, neonatal unit; PICU, paediatric intensive care unit.

In total, over 4 million babies were born from 1 January 2013 to 31 December 2018, of whom 384 747 babies were admitted to an NNU for at least 1 day of specialist neonatal care in the first week of life in England and Wales (figure 1). In total, 37 549 children born in the same time window were admitted to PICU from 1 January 2013 to 31 December 2020 before the age of 2 years.

### PICU admissions as a proportion of entire cohort of admissions to NNU

**Figure 2:**
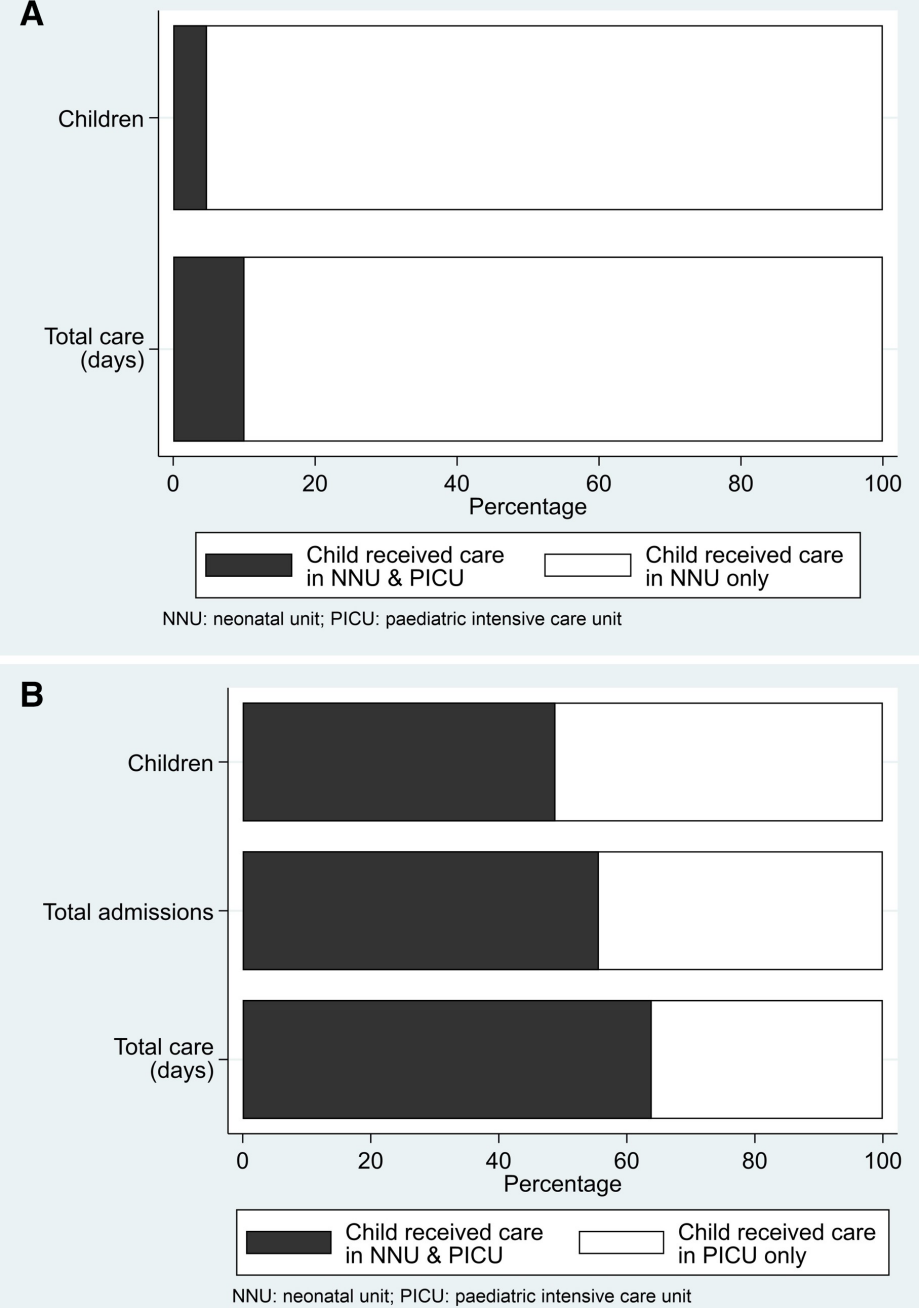
Percentage of workload contribution in the NNU and PICU for those children who received neonatal care after birth. (A) Percentage of neonatal care accounted for by children who were admitted to a PICU in the first 2 years of life born from 2013 to 2018. (B) Percentage of PICU care provided to children who received neonatal care out of all PICU children/admissions <2 years born from 2013 to 2018.

The majority of babies admitted to an NNU were not admitted to PICU: 18 343 (4.8%) experienced at least one PICU admission (figure 2A). There were 2769 (0.7%: 2769/384 747) children who experienced at least one PICU admission before returning to NNU to receive ongoing care, 4381 children had their first PICU admission as a direct transfer from NNU and the remainder had their first admission sometime later (figure 1). Information about the children by the source of their first PICU admission is provided in online supplemental table 1.

10.1136/archdischild-2023-325986.supp1 Supplementary data

Extremely preterm babies had the highest proportion of PICU admissions with 13.4% (n=2020/14 547, table 1) admitted to PICU at least once. This risk broadly reduced with increasing gestational age, with 4.7% (9591/202 566, table 1) of babies born at term who required an NNU admission were also admitted to PICU. Babies who received at least 1 day of intensive care in NNU had the highest percentage of PICU admission (11.6%) and for those with a maximum level of care of special care this was 2.2% (table 1).

**Table 1:** All babies born and admitted to neonatal care broken down by those who received care in NNU and PICU (n=18 343) and those who who received care in NNU only (n=3 66 404)

	Admitted to paediatric intensive care (n=18 343)	Not admitted to paediatric intensive care (n=366 404)	Total (n=384 747)
Gestational age, n (%)			
<28 weeks	2020 (13.4)	12 527 (86.1)	14 547
28–31 weeks	2035 (6.3)	30 114 (93.7)	32 149
32–36 weeks	4697 (3.5)	130 761 (96.5)	135 458
37+ weeks	9591 (4.7)	192 975 (95.3)	202 566
Missing	–	27 (100)	27
Sex, n (%)			
Male	10 808 (4.9)	208 384 (95.1)	219 192
Female	7485 (4.5)	157 750 (95.5)	165 235
Unspecified/missing	50 (15.6)	270 (84.4)	320
Maternal ethnicity, n (%)			
White	11 209 (4.6)	230 599 (95.4)	241 808
Mixed	228 (4.7)	4594 (95.3)	4822
Asian or Asian British	2205 (5.6)	36 890 (94.4)	39 095
Black or Black British	1083 (5.7)	17 964 (94.3)	19 047
Other	338 (4.8)	6758 (95.2)	7096
Missing	3280 (4.5)	69 599 (95.5)	72 879
Deprivation quintile, n (%)			
Most deprived	4832 (5.5)	82 698 (94.5)	87 530
2	3362 (4.8)	66 753 (95.2)	70 115
3	2633 (4.7)	53 789 (95.3)	56 422
4	2078 (4.3)	46 025 (95.7)	48 103
Least deprived	1744 (4.3)	39 163 (95.7)	40 907
Missing	3694 (4.5)	77 976 (95.5)	81 670
Birth weight in grams, mean (SD)*	2466 (1009)	2719 (934)	2707 (939)
Multiplicity, n (%)			
Singleton	16 258 (4.9)	317 058 (95.1)	333 316
Twins or higher order	2084 (4.1)	49 268 (95.9)	51 352
Missing	1 (1.3)	78 (98.7)	79
Highest level of neonatal care, n (%)			
Intensive care	11 685 (11.6)	88 721 (88.4)	100 406
High dependency care	2319 (2.8)	80 430 (97.2)	82 749
Special care	4339 (2.2)	197 253 (97.9)	201 592
Days at each level of neonatal care, median (25th to 75th centile)			
Intensive care	5 (2 to 15)	4 (2 to 8)	4 (2 to 9)
High dependency care	8 (2 to 30)	2 (1 to 6)	3 (1 to 7)
Special care	9 (3 to 22)	4 (2 to 12)	4 (2 to 13)

Percentage is of the row total.

*Note: 50 cases of missing birth weight.

NNU, neonatal unit; PICU, paediatric intensive care unit.

### PICU admissions aged under 2 years of age who have previously been care for in NNU

The largest group of PICU admissions were for cardiac conditions (n=7138), the majority of whom were born at term (n=5146, table 2). These children had the shortest NNU stay, likely reflecting the early transfer to a cardiac centre as part of a planned care pathway. This contrasts with preterm babies with a cardiac condition who had the longest median NNU stay.

**Table 2:** Characteristics by clinical reason for first admission to PICU (n=18 343) for those children admitted to neonatal care and PICU

	All first admissions (n=18 343)	First admission to PICU was for cardiac reasons born preterm (n=1992)	First admission to PICU was for cardiac reasons born at term (n=5146)	First admission to PICU was for respiratory reasons (n=5386)	First admission to PICU was for gastrointestinal reasons (n=1893)	First admission to PICU was for neurological reasons (n=939)	First admission to PICU was for other reasons (n=2987)
Gestational age, n (%)							
<28 weeks	2020 (11.0)	360 (18.1)	N/A	782 (14.5)	394 (20.8)	129 (13.7)	355 (11.9)
28–31 weeks	2035 (11.1)	305 (15.3)	N/A	987 (18.3)	244 (12.9)	111 (11.8)	388 (13.0)
32–36 weeks	4679 (25.6)	1327 (66.6)	N/A	1781 (33.1)	428 (22.6)	262 (27.9)	899 (30.1)
37+ weeks	9591 (52.3)	N/A	5146 (100)	1836 (34.1)	827 (43.7)	437 (46.5)	1345 (45.0)
Neonatal length of stay, median (IQR)	11 (3–40)	32 (9–82)	3 (1–7)	22 (8–56)	18 (3–65)	18 (4–52)	14 (3–45)
Postnatal age on first admission to PICU (days), median (IQR)	43 (5–139)	65 (22–147)	8 (1–88)	73 (32–177)	9 (2–71)	82 (23–342)	44 (3–143)
Length of first PICU stay, median (IQR)	5 (3–8)	4 (2–7)	5 (3–8)	6 (4–9)	3 (2–6)	4 (2–7)	4 (2–8)
Total PICU stay over first 2 years, median (IQR)	6 (3–14)	6 (3–16)	8 (4–16)	7 (4–14)	4 (2–8)	5 (2–10)	6 (3–12)
First admission was emergency, n (%)	11 016 (60.1)	713 (35.8)	1924 (37.4)	4564 (84.7)	1239 (65.5)	678 (72.2)	1898 (63.5)
First PICU admission was during neonatal care, n (%)	2769 (15.1)	546 (27.4)	308 (6.0)	485 (9.0)	769 (40.6)	183 (19.5)	478 (16.0)
>1 PICU admission, n (%)	6177 (33.7)	728 (36.6)	2360 (45.9)	1514 (28.1)	494 (26.1)	268 (28.5)	813 (27.2)

Percentage is of the column total.

PICU, paediatric intensive care unit.

The second largest group of PICU admissions (n=5386) were children with respiratory conditions, of which 65.9% were preterm (table 2), with a median age on first PICU admission of 73 days. Respiratory conditions were also the main reason for PICU admission of preterm-born children (online supplemental figure 1) and most of the first admissions were an emergency (84.7%, table 2).

Babies admitted to PICU during their neonatal stay with a readmission to NNU were mainly admitted for cardiovascular (eg, patent ductus arteriosus) or gastrointestinal (eg, necrotising enterocolitis) reasons (online supplemental table 1). Conversely, children admitted to PICU sometime after their neonatal stay were primarily admitted for cardiovascular problems (~80% planned) or respiratory problems (~90% emergency).

### Multiple PICU admissions for children previously cared for in NNU

**Figure 3:**
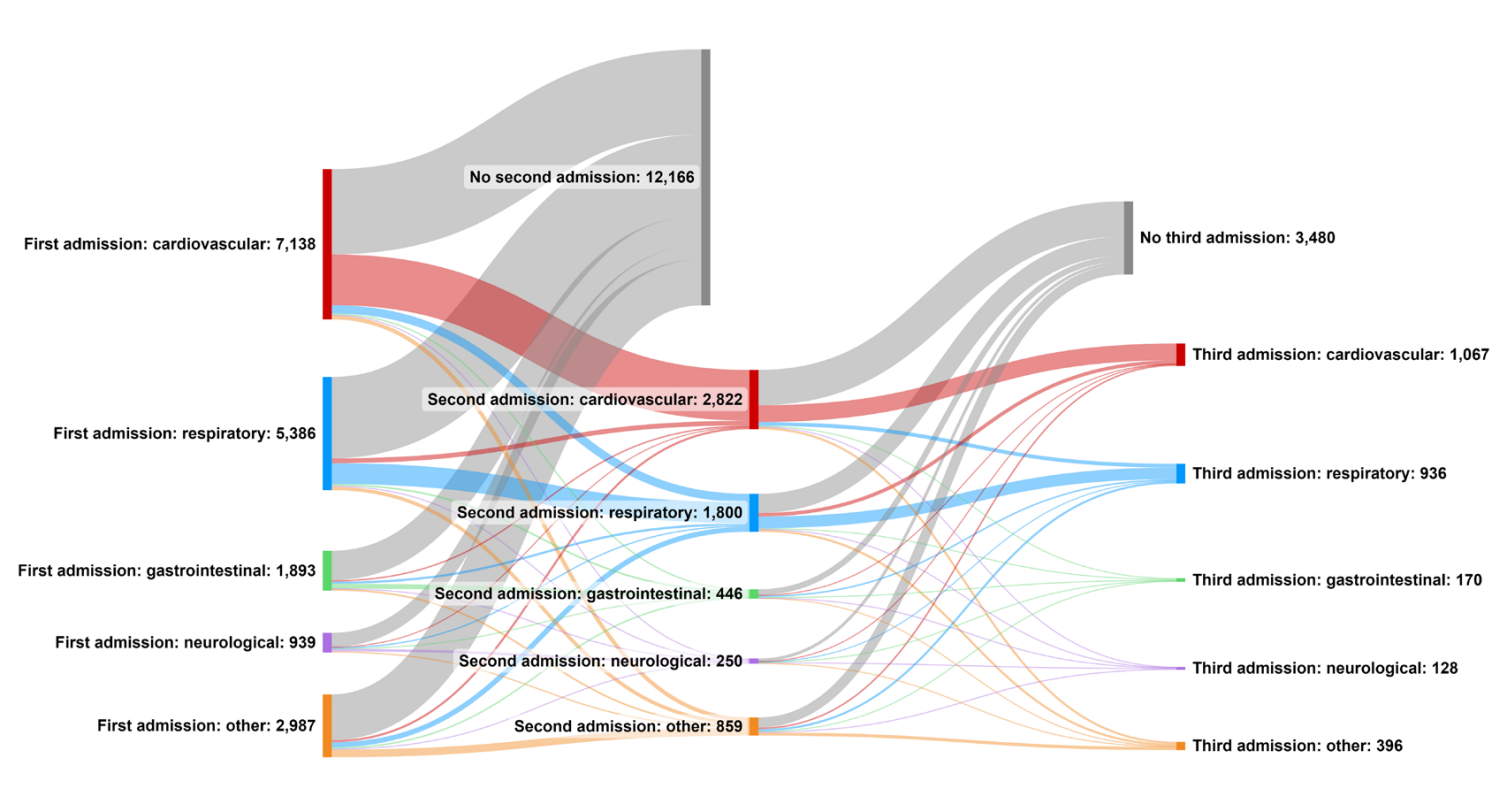
Sankey graph to show reasons for repeated admissions to paediatric intensive care unit.

A third of the 18 343 children had more than one PICU admission in the first 2 years of life (table 2). Most second admissions to PICU were for cardiac reasons (n=2822, 45.7% of all second admissions), likely reflecting the planned care pathway for those children undergoing planned staged surgical repairs (figure 3). However, a large proportion of second admissions (n=1800, 29.1% of all second admissions) were for respiratory reasons, including children who had been admitted previously to PICU for other reasons.

### Admissions to PICU of children previously cared for in NNU as a proportion of entire cohort of admissions to PICU under the age of 2 years

There were 37 549 children (55 093 admissions) born between 1 January 2013 and 31 December 2018 admitted to a PICU before the age of 2 years (figure 1). The 18 343 children who had been cared for in NNU accounted for 48.9% of the children in PICU, 55.6% of the PICU admissions and over 60% of all PICU care days for those aged less than 2 years (figure 2B).

## Discussion

Our study has quantified the characteristics and workload of children admitted to both NNU and PICU in the first 2 years of life. Within the general population, only 1% of children aged <1 year are admitted to PICU, but of those admitted to NNU, 4.8% were also admitted to PICU before 2 years. While only a small percentage of babies admitted to NNU required admission to PICU, these children represent approximately half of the children admitted to PICU aged under 2 years. Given approximately 40% of all admissions to PICU are from children aged under 1 year,^6^ those previously cared for in an NNU represent a large proportion of the overall PICU workload. Therefore, while both specialities see some of the same children at similar time points in early life, each has a very different perspective due to the proportion of workload that these children contribute.

Respiratory and cardiac conditions accounted for nearly 70% of all first PICU admissions from children previously cared for in NNU. The largest group was cardiac conditions who may be following a planned care pathway following the antenatal diagnosis of a cardiac anomaly,^12^ with 60% of first cardiac PICU admissions being elective. Most of these children were born at term and had short NNU stays. Cardiac conditions were also responsible for nearly half of all second PICU admissions showing that ongoing care needs and admissions may increase due to improved survival of children with complex conditions such as hypoplastic left heart syndrome.^13^

Respiratory admissions were the second most common reason for PICU admission and in absolute numbers made up a similar number of first PICU admissions to term cardiac babies. These children are likely to have PICU workload implications as most respiratory admissions were emergency admissions, and these are more common in winter when the healthcare service is under increased pressure.^14^ These children were more likely to have been born preterm and therefore at higher risk of respiratory conditions such as bronchiolitis and were older on admission to PICU. Other work from our group has explored the risk of PICU admission for very preterm-born children further.^15^

Care pathways within the UK may differ from other countries, demonstrated by the babies who were admitted to PICU during their NNU stay (eg, for necrotising enterocolitis surgery). Therefore, these findings are specific to the UK where some NNUs are unable to provide surgery and may not be generalisable.

Readmission rates to PICU were high across our cohort with one-third of children being readmitted to PICU at least once. This rate of readmission was seen across all diagnostic groups, indicating it was not entirely accounted for by planned multiple admissions for elective surgery (eg, staged cardiac surgery) although cardiac admissions did have the highest rate of PICU readmission. Our population seems similar to medically complex children where PICU readmission rates of 20–30% have been observed.^16 17^

Across the entire population of children aged <1 year, the percentage admitted to PICU is ~1%.^6^ Our population of children who had been in NNU had a higher risk of admission to PICU and this persisted even for those receiving lower levels of NNU care, suggesting they are children with increased medical need. While some children transitioning from NNU to PICU follow an established care pathway, these findings suggest increased need for collaboration between and across neonatal and paediatric critical care services to effectively plan for the potentially complex needs of children and their families.

### Strengths and limitations

Our work is unique, as while small studies have investigated hospitalisation of children discharged from NNUs in specific populations, such as those born very preterm,^18 19^ or children admitted to PICU during (but not after) their neonatal stay, to the best of our knowledge, this is the first study to link NNU and PICU data on a population level for individual children. The NNRD and PICANet are national data sources with high levels of ascertainment, completeness and data quality including for personal identifiers, providing potential for high levels of linkage success, although we cannot quantify how many children, if any, were missed.

A limitation of our work is the PICU admissions included some children who received high dependency care, which was still provided in a PICU setting and thus contributed to PICU workload, but this may affect the generalisability of our results internationally. Where we have broken down the admission source, this is an approximation as we do not have exact dates or times of care. While combining this group creates a heterogeneous cohort, together they contribute a large part of the PICU workload provided to young children and we felt it was vital to quantify the whole population. In future work, we will look at different pathways leading to PICU admission and explore healthcare in other settings such as children’s wards via linkage to other data (eg, Hospital Episodes Statistics).

A limitation of our work is that we were not able to present detailed reasons for PICU admission as the population is heterogeneous and the various diagnoses are many and varied. Therefore, we grouped together diagnoses into established categories, for example, ‘respiratory’ within which the most common diagnosis was bronchiolitis. While this is a limitation, we believe it important for this cohort to be considered in its entirety in terms of workload for both neonatal and paediatric communities.

### Future work and conclusions

We have focused on the characteristics of children needing care in NNU and PICU. Future work will focus on specific clinical subgroups of children (eg, those born very preterm or congenital anomalies or neurological conditions) to explore risk factors, including child demographics, for PICU admission, with focus on unplanned PICU admissions and their timing, which may cause the most stress for families and unanticipated demand on healthcare services.

The healthcare professionals caring for babies and children in NNUs and PICUs see some of the same children, and therefore, there is a need for close working relationships to ensure smooth transitions for those children who may potentially have ongoing healthcare needs.

## Data Availability

Data may be obtained from a third party and are not publicly available. Data may be obtained from a third party and are not publicly available. PICANet data may be requested from the data controller, the Healthcare Quality Improvement Partnership (HQIP). A Data Access Request Form can be obtained from https://www.hqip.org.uk/national-programmes/accessing-ncapop-data/%23.XQeml_lKhjU
