# Supplementary figures and images for: Characteristics of children requiring admission to neonatal care and paediatric intensive care before the age of 2 years in England and Wales: a data linkage study
Source: Arch Dis Child. 2024 Feb 12;109(5):387–94. doi: 10.1136/archdischild-2023-325986 (PMC11041614; doi:10.1136/archdischild-2023-325986)

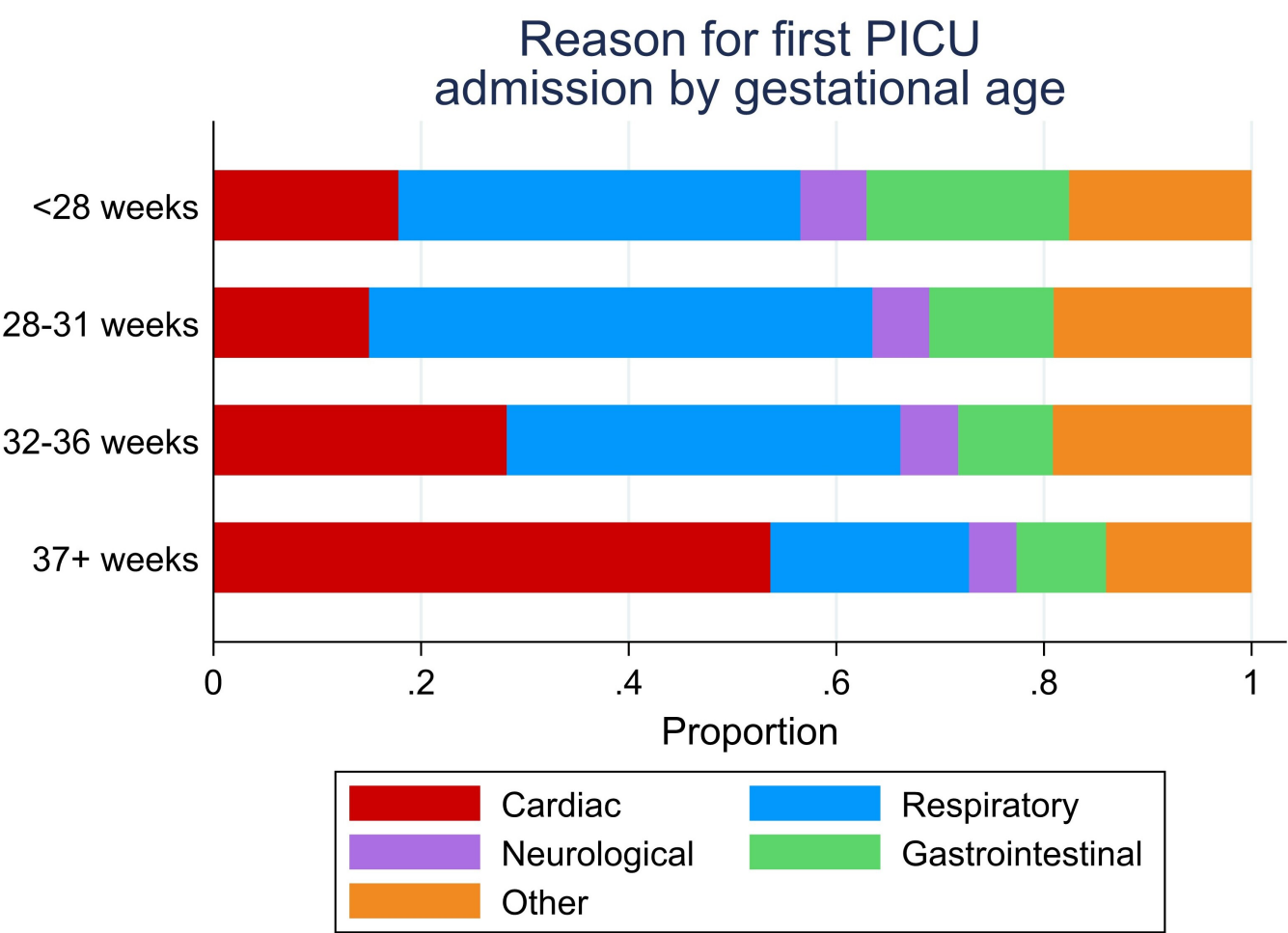

Supplement: Supplementary data [file archdischild-2023-325986supp001.pdf]
